# Identification of Functional Determinants in the Chikungunya Virus E2 Protein

**DOI:** 10.1371/journal.pntd.0005318

**Published:** 2017-01-23

**Authors:** Christopher Weber, Eva Berberich, Christine von Rhein, Lisa Henß, Eberhard Hildt, Barbara S. Schnierle

**Affiliations:** Paul-Ehrlich-Institut, Department of Virology, Paul-Ehrlich Strasse, Langen, Germany; University of Texas Medical Branch, UNITED STATES

## Abstract

**Background:**

Chikungunya virus (CHIKV) is a mosquito-transmitted alphavirus that causes high fever, rash, and recurrent arthritis in humans. It has efficiently adapted to *Aedes albopictus*, which also inhabits temperate regions, including Europe and the United States of America. In the past, CHIKV has mainly affected developing countries, but has recently caused large outbreaks in the Caribbean and Latin America. No treatment or licensed CHIKV vaccine exists.

**Methodology/Principal Findings:**

Here, we have identified determinants in the CHIKV cell-attachment protein E2 that facilitate cell binding. The extracellular part of the E2 gene is subdivided into the three domains, A, B, and C. These domains were expressed in *E*. *coli* and as Fc-fusion proteins generated from HEK293T cells and used for cell-binding assays. Domains A and B bound to all cells tested, independently of their permissiveness to CHIKV infection. Domain C did not bind to cells at all. Furthermore, CHIKV cell entry was promoted by cell-surface glycosaminoglycans (GAGs) and domain B interacted exclusively with GAG-expressing cells. Domain A also bound, although only moderately, to GAG-deficient cells. Soluble GAGs were able to inhibit CHIKV infection up to 90%; however, they enhanced the transduction rate of CHIKV Env pseudotyped vectors in GAG-negative cells.

**Conclusion/Significance:**

These data imply that CHIKV uses at least two mechanisms to enter cells, one GAG-dependent, via initial attachment through domain B, and the other GAG-independent, via attachment of domain A. These data give indications that CHIKV uses multiple mechanisms to enter cells and shows the potential of GAGs as lead structures for developing antiviral drugs.

## Author Summary

The chikungunya virus (CHIKV) glycoprotein E2 mediates cell attachment and consists of three domains A, B and C. Since the cell entry process of CHIKV is not understood in detail, we analyzed the binding properties of the three E2 domains with proteins expressed in *E*. *coli* or as Fc-fusion proteins and the role of glycosaminoglycans (GAGs) on E2 cell binding and CHIKV entry. The two surface-exposed E2 domains, A and B, both bound to cells and domain B bound only to cells expressing GAGs. Domain A bound additionally to GAG-deficient cells and domain C did not bind to cells. CHIKV-pseudotyped lentiviral vector and CHIKV entry were enhanced in cells expressing GAGs. Our results suggest that CHIKV uses at least two entry mechanisms, one GAG-dependent, via attachment through E2 domain B, and the other GAG-independent, via binding of domain A. These data give indications that CHIKV uses multiple mechanisms to enter cells and shows the potential of GAGs as lead structures for developing antiviral drugs. In addition, it shows that domain A and B might constitute good targets for vaccine development.

## Introduction

The Chikungunya virus (CHIKV) is a mosquito-transmitted alphavirus that causes high fever, rash, and recurrent arthritis in humans. The majority of symptoms disappear after about one week. However, in about 30% of cases, arthritis can last for months or even years, which may cause substantial economic losses [1], [2]. The virus has been endemic in Sub-Saharan Africa, the Indian Ocean islands, India, and Southeast Asia. However, the virus spread to the Caribbean in late 2013 and is now responsible for a large, still-ongoing outbreak there and in Latin America with 1.9 million suspected cases as of December 2016 (www.paho.org/hy/). The mortality rate is very low (0.1%), but the infection rates are high (sometimes 30%) and asymptomatic cases are rare (about 15%). Due to climate change, globalization, and vector switching, the virus will most likely continue to cause new, worldwide outbreaks. Additionally, more temperate regions of the world like Europe or the USA, which have recently reported their first cases, will likely become targets [3], [4]. Alarmingly, no specific treatment or vaccination against CHIKV is available so far.

CHIKV is a (+) single-stranded RNA virus. Like other alphaviruses, it enters cells by receptor-mediated endocytosis and a subsequent pH-dependent fusion step. CHIKV has two surface proteins that mediate cell entry: the transmembrane glycoproteins E2 and E1. E2 mediates cell attachment and E1 is a class II viral fusion protein [5], [6]. E2 and E1 associate as trimers of heterodimers (E2–E1) on the particle surface [7], [8], [9]. The E2 protein contains two N-glycosylation sites at position 263 and 345. The E2 envelope protein consists of domain C, located close to the viral membrane, domain A, in the center of the protein, and domain B, at the distal end, prominently exposed on the viral surface [7], [8]. These domains are promising sites of interaction with the target cell.

Potential interaction partners of viruses on the cell surface are glycosaminoglycans (GAGs), which are ubiquitously present on the surfaces of all animal cells and are an essential part of the extracellular matrix (ECM) [10],[11], [12]. They consist of long linear chains of disaccharide units (30–60 per chain). These disaccharides are sulfated to different degrees and are thus negatively charged. GAGs that are covalently linked to a core protein are called proteoglycans (PGs). They differ depending on the carbohydrates that form the disaccharide units. The best characterized GAGs linked to core proteins on human cells are heparan sulfate (HS), chondroitin sulfate (CS), and dermatan sulfate (DS) [12]. Since GAGs are ubiquitously present on the cell surface, many pathogens exploit them to cross the cell membrane barrier and use them for initial cell attachment or as entry receptors. These pathogens include several bacteria, parasites, and viruses [10], [13]. Cell surface HS, the most extensively studied GAG, promotes attachment and/or entry of herpes simplex virus type 1 (HSV-1), human immunodeficiency virus (HIV), hepatitis C virus (HCV), vaccinia virus (VACV), dengue virus (DENV), and adeno-associated virus isolate 2 (AAV-2) into cells [13]. Binding of an alphavirus, the eastern equine encephalitis virus (EEEV), to cell surface HS, thereby enhancing its neurovirulence, has also been reported [14]. So far, the role of GAGs in CHIKV replication has only been studied in the context of viral attenuation. Point mutations within domain A of the E2 protein (e.g., E79K or G82R) have been found in attenuated vaccine strains that were cell culture adapted and showed enhanced GAG dependency but reduced *in vivo* replication [15], [16], [17], [18]. Additionally, it was reported that cell-surface PGs promote replication of some CHIKV strains, but this replication was not inhibited by the presence of soluble GAGs [15]. Furthermore, one CHIKV strain has been shown to be not influenced at all by the presence or absence of PGs [17]. The E2 domains A and B have been suggested before to be putative receptor binding sites [7], [8].

Since the cell entry process of CHIKV is not understood in detail [19], we examined the role of GAGs in CHIKV cell entry and analyzed the binding properties of the E2 domains A, B and C, and their dependency on GAGs. The two surface-exposed E2 domains, A and B, both bound to cells expressing GAGs. Domain C was not involved in cell binding at all. We could show that CHIKV entry is enhanced in cells expressing GAGs and that domain B binds exclusively to GAGs. Domain A also bound to cells that do not express GAGs. Our results suggest that CHIKV uses at least two entry mechanisms, one GAG-dependent, via attachment through E2 domain B, and the other GAG-independent, via domain A.

## Materials and Methods

### Cell culture

All cells used for this study were cultured at 37°C under 5% CO_2_. HEK 293T (ATCC: CRL-1573) cells were incubated in Dulbecco’s modified Eagle’s medium (DMEM; Lonza, Verviers, Belgium). Jurkat (ATCC: TIB-152) and BHK-21 (CCL-10) cells were grown in Roswell Park Memorial Institute medium (RPMI; Biowest, Nuaille, France). CHO-K1 and pgsA-745 cells were grown in Ham’s F-12 medium (Life technologies, Darmstadt, Germany) and 2 mM glutamine. Media were supplemented with 10% FBS (v/v; PAA, Pasching, Austria) and 5% L-glutamine (200 mM; Lonza, Verviers, Belgium). The glycosaminoglycans chondroitin sulfate, dermatan sulfate, heparan sulfate, heparin and dextran sulfate were purchased from Sigma-Aldrich (Taufkirchen, Germany).

### Plasmids

The codon-optimized CHIKV E3-E1 gene (based on isolate “S27”) was synthesized by GeneArt (Life Technologies, Darmstadt) and cloned into the plasmid pIRES2-eGFP (Clontech/Takara, Saint-Germain-en-Laye, France) as described previously [20].

The E2 domain A (including the β-ribbon connector), B (aa 172–231), and C (aa 271–341) genes were cloned into the bacterial expression vector pET-15b. The same was done with the entire extracellular part of the E2 protein (E2ex). Cloning was achieved by adding the restriction sites *NdeI* and *Bam*HI via primers by PCR (template DNA: pIRES2-EGFP-CHIKV E3-E1), cutting the DNA products and the vector, and subsequent ligation. For domain A, two fragments were derived via PCR. One fragment contained domain A itself and the first part of the β-ribbon connector (C-terminus of domain A (aa 1–171)). The other fragment contained the second half of the connector C-terminus of domain B (aa 231–270). The fragments were cloned into the pET-15b vector by a triple ligation (via *Nde*I and *Bam*HI). The two fragments were linked via a shared *Sma*I restriction site (at the C-terminal part of fragment one and the N-terminal part of fragment two, respectively). By this procedure, E2 domain B was bypassed and replaced with the sequence G_4_PG_5_. The constructs also contained an N-terminal poly-histidine-tag for purification.

The primers used for cloning were:

Domain E2ex: Dom AI fw 5’-AAAACATATGAGCACCAAGGACAACTTCAAC, Dom C rev 5’-AAAAGGATCCTCACTGGGGCCAGTACTTGTAGG;
Domain A: Dom AI fw 5’-AAAACATATGAGCACCAAGGACAACTTCAAC, Dom AI rev 5’-AAAACCCGGGGCCGCCGCCGCCGGGCATGTGCACCTCGATC, Dom AII fw 5’-AAAACCCGGGGGCGGCGGCGGCAACCACAAGAAGTGGCAGTAC, Dom AII rev 5’-AAAAGGATCCTCACTTGGGCACCATGCAGGTC;
Domain B: Dom B fw 5’-AAAACATATGCCCGACACCCCCGATAGAA, Dom B rev 5’-AAAAGGATCCTCAGGTCACGGCGGCGTGGC;
Domain C: Dom C fw 5’-AAAACATATGGCCCGGAACCCTACCGTG, Dom C rev 5’-AAAAGGATCCTCACTGGGGCCAGTACTTGTAGG).

The Fc-fusions were constructed by cloning E2 fragments via Apa I, Nhe I sites introduced by PCR, into the vector pCMV2.5-hIgG1Fc-XP (kind gift of Stephan Dübel, TU Braunschweig). This generated expression vectors that express E2-Fc fusion proteins without any tags. The following primers were used to amplify the E2 fragments from the *E*.*coli* expression vectors as template: Domain A fw 5’ AAAAGGGCCCAGCACCAAGGACAACTTCAAC, rev 5’AAAAGCTAGCCTTGGGCACCATGCAGGTC; domain B fw 5’ AAAAGGGCCCCCCGACACCCCCGATAGAA, rev 5’AAAAGCTAGCGGTCACGGCGGCGTGGC; domain C fw 5’ AAAAGGGCCCGCCCGGAACCCTACCGTG, rev 5’AAAAGCTAGCCTGGGGCCAGTACTTGTAGG; domain A-ß fw 5’ AAAAGGGCCCAGCACCAAGGACAACTTCAAC, rev 5’AAAAGCTAGCGGGGTCGTGGTGGAAGGG. All mutations were introduced by site directed mutagenesis.

### Protein expression und purification

Proteins were expressed in BL21-CodonPlus (DE3)-RIPL competent cells (Agilent Technologies, Böblingen, Germany) transformed with the pET-15b plasmid containing construct A, B, C, or E2ex. Bacteria were inoculated into 100 ml of LB medium containing ampicillin (0.1 mg/ml) and grown overnight (37°C, 220 rpm). After 16 hrs, 2 l of LB medium were inoculated with the 100 ml overnight culture. The bacteria were grown to an OD_600_ of 0.5–0.7, and then protein expression was induced by the addition of 1 mM IPTG. After another 2.5 hrs of incubation, cells were harvested and the pellets were frozen at –20°C.

The recombinant proteins were purified from the bacterial pellets under native (B, C) or denaturing (A, E2) conditions using HisTrap FF Crude columns (GE Healthcare, Freiburg, Germany) and the ÄKTA system (GE Healthcare, Freiburg, Germany) as described by [21]. For A and E2, ion-exchange chromatography was additionally performed to remove contaminating bacterial proteins. After purification, proteins were dialyzed against PBS using Slide-A-Lyzer Dialysis Cassettes 3.5K MWCO (Pierce, Thermo Scientific, Bonn, Germany) and concentrated with Ultra-4 3 kDa Centrifugal Filter Units (Merck Millipore, Schwalbach, Germany). The protein concentration was determined by SDS-PAGE with marker proteins, following staining with Coomassie (Bio-Rad, Munich, Germany). Proteins were then quick-frozen with liquid nitrogen and stored at –80°C. For experiments, proteins were thawed in a 37°C water bath.

### Fc-fusion protein expression and purification

The empty Fc control protein and the E2 domain-Fc-fusion proteins were produced in HEK293T cells by transient transfections. Transiently transfected HEK293T cells were grown in DMEM containing 10% FCS. After 48 h, supernatants of the transfected cells were harvested two times at 24 h intervals. The secreted Fc-fusion proteins were purified by affinity chromatography with protein A-agarose, eluted at a pH 2.5, neutralized with 1M Tris, pH 9.0 and dialyzed against PBS pH 7 and stored at -80°C.

### Production of CHIKV-mCherry-490 and infection

The plasmid pCHIKV-mCherry-490 [22] was *in vitro*-transcribed with T7 RNA polymerase after *Not*I linearization. The mRNA was transfected into BHK-21 cells using Lipofectamine 2000 (according to the manufacturer’s protocol; Life Technologies). Virus-containing supernatants were harvested 48 hrs later and used to reinfect fresh BHK-21 cells for virus amplification. Infected cells showed a clear red fluorescence. Supernatants were collected and stored at –80°C or used to determine the viral titer.

For CHIKV infections, 293T cells were seeded onto a 24 well plate. Cells were incubated at 37°C for 16–24 hrs and counted (about 3 × 10^5^ cells). Subsequently, the mCherry-tagged CHIKV (CHIKV-mCherry-490) [22] was added at a multiplicity of infection (MOI) of 1. After 6 hrs, the cells were collected in medium, washed and resuspended in 2% paraformaldehyde in PBS, and analyzed by flow cytometry. At least 10,000 events were acquired with an LSRII instrument (BD Biosciences) and analyzed using FACS Diva software.

### Lentiviral vector particle production

Lentiviral vector particle production was performed as described previously [20]. Briefly, 293T cells were seeded in 10 cm dishes in 10 ml DMEM. Cells were cotransfected 16 hrs post seeding with the plasmids pRRLsinhCMV-GFP-pre (a lentiviral vector genome encoding GFP) or pCSII-Luc (a lentiviral vector genome encoding luciferase), pMDLg/pRRE, pRSVrev, and pHIT-G or pIRES2-eGFP-CHIKV E3-E1 using Lipofectamine 2000 (according to the manufacturer’s protocol; Life Technologies). After 24 hrs incubation, the medium was discarded and replaced with 5 ml of fresh DMEM. Another 24 hrs later, the supernatant containing the vector particles was harvested, sterile filtered with 0.45 μm filters (Sartorius, Göttingen, Germany), and frozen at -80°C.

### Transduction of cells with lentiviral vector particles

Cells were transduced with pseudotyped lentiviral vector particles in 384-well plates as described previously [20]. Briefly, 6000 293T cells per well were seeded (using a MultiFlo Microplate Dispenser; BioTek, Bad Friedrichshall, Germany) in 20 μl DMEM in white CELLSTAR 384-well microtiter plates (Greiner Bio-One, Frickenhausen, Germany) and incubated for 16–24 hrs at 37°C. The same was done for CHO-K1 and pgsA-745 cells using Ham’s F-12 medium and 3000 cells per well were seeded.

Soluble glycosaminoglycans (GAGs) were serially diluted (three-fold) four times in DMEM containing vector particles in 96-U-well plates (Thermo Scientific, Rockford, IL, USA), and incubated at 4°C for 1 h. This resulted in equal amounts of vector and serially diluted compound. The vector particle mixtures were then added to the cells using a Matrix Multichannel Equalizer Electronic Pipette (Thermo Scientific, Rockford, IL, USA), transferring 20 μl each to three wells of the 384-well plate out of one well of the 96-well plate. This resulted in a final concentration of GAGs/dextran sulfate ranging from 500 to 6.2 μg/ml. Cells were incubated with the vector particle mixtures for another 16–24 hrs. Afterwards, 20 μl of BriteLite substrate (PerkinElmer, Rodgau, Germany) was added. After 5 minutes incubation at room temperature, the luciferase signal was detected using the PHERAstar FS microplate reader (BMG LABTECH, Ortenberg, Germany).

### Statistical analysis

Statistical analyses were done using the GraphPad Prism 5.04 software (La Jolla, CA, USA). The p-values were determined by the unpaired two-tailed *t*-test.

### Protein cell-binding assay

The recombinant proteins purified from *E*. *coli* or E2-Fc-fusion proteins were incubated for 30 min at 4°C with cells in PBS/2% FCS. The cells were then washed and bound protein was detected via an anti His-tag antibody (Dianova, Hamburg, Germany) and an anti-mouse IgG-FITC antibody or an FITC coupled anti-human IgG antibody (Fc-fusion proteins) followed by flow cytometry. Binding was detected as the mean change in fluorescence. In addition, the CHIKV E2-derived protein sA (containing the surface exposed regions of domain A connected by linkers) [23] was used as a negative control for the binding of *E*.*coli* derived proteins and the Fc-protein served as negative control for Fc-fusion proteins and the values are given as fold increase in binding compared to Fc.

## Results

### CHIKV cell entry is enhanced by glycosaminoglycans (GAGs)

**Fig 1:**
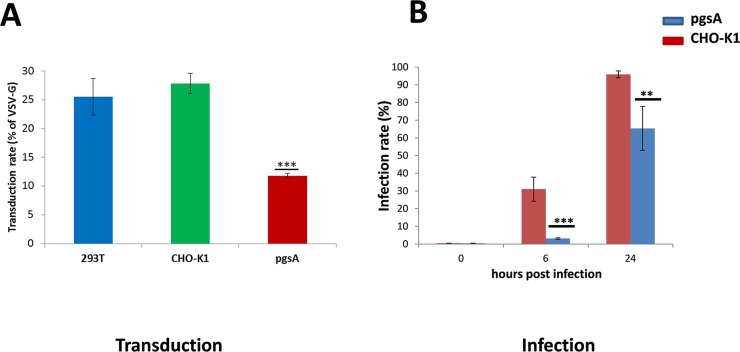
Transduction or infection of cells with CHIKV Env pseudotyped vector particles. A: 293T, CHO-K1, and pgsA-745 cells were seeded in 24-well plates and transduced with *gfp*-encoding CHIKV Env-pseudotyped lentiviral vector particles. The cells were analyzed by flow cytometry for GFP expression 48 hrs post transduction. The proportion of GFP-positive cells is given relative to the proportion of positive cells following transduction with VSV-G-pseudotyped vectors (control). Data represent the average of three independent experiments. *** (P ≤ 0.001) indicates significant differences in transduction rates compared to CHO-K1 cells. B: CHO-K1 and pgsA-745 cells were seeded in 24-well plates and infected with CHIKV-mCherry at an MOI of 1. The viral replication was determined 6 and 24 hrs post-infection, respectively, by flow cytometry detecting mCherry. Data represent the average of three independent experiments. ** and *** indicate significant differences in infection rates to CHO-K1 cells after 6 and 24 hrs, respectively. * (P ≤ 0.05) and ** (P ≤ 0.01).

First it was analyzed if GAGs play a role in viral entry. For this investigation, 293T cells, CHO-K1 cells, and the CHO-K1 derived cell line pgsA-745 which, due to an enzymatic defect, is not able to produce GAGs, were transduced with CHIKV Env- or VSV-G-pseudotyped lentiviral vectors encoding GFP. Transduction was determined as the number of GFP-positive cells and was standardized as percentage of GFP-positive cells obtained after transduction with VSV-G-pseudotyped vectors. The results displayed in Fig 1A show that CHIKV cell entry into 293T and CHO-K1 cells was almost equally efficient. However, the transduction rate of pgsA-745 cells was significantly reduced by more than 50% in comparison to the parental cell line. Thus, cell entry of CHIKV Env-pseudotyped vectors into GAG-deficient cells is strongly and significantly reduced in comparison to those carrying cell-surface GAGs. However, cell entry was only reduced by about 50%, indicating the existence of at least one more entry pathway.

To confirm the relevance of the above experiments, the dependency of CHIKV infections on cell-surface GAGs was studied. For this, CHO-K1 and pgsA-745 cells were both infected with the recombinant CHIKV-mCherry-490 using an MOI of 1. This virus contains an mCherry gene within the nsP3 gene of CHIKV and has growth characteristics similar to the wild-type virus [22], [24]. Viral replication was determined at 6 and 24 hrs post-infection by flow cytometry. The infection rate at 6 hrs post-infection was significantly reduced (9.9-fold) in pgsA-745 cells compared to CHO-K1 cells (Fig 1B). At 24 hrs post-infection, the difference decreased to a 1.5-fold higher, yet still significantly different, infection rate in CHO-K1 cells compared to pgsA-745 cells. Thus, both the cell entry of CHIKV Env-pseudotyped vectors and the replication of CHIKV were reduced on pgsA-745 cells lacking cell-surface GAGs, but not fully inhibited, indicating that GAGs enhance, but are not essential for entry.

### Transduction with CHIKV Env-pseudotyped vector particles and infection of cells with CHIKV is inhibited by soluble GAGs

CHIKV cell entry into GAG-deficient cells was reduced; accordingly, the presence of soluble GAGs might inhibit CHIKV entry into GAG-expressing cells or influence entry into GAG-free cells. Therefore, 293T, CHO-K1, and pgsA-745 cells were transduced with CHIKV Env-pseudotyped vectors in the presence of different amounts of soluble GAGs. Dextran sulfate (DX), which consists of long chains of highly sulfated glucose units, was used as a control for the soluble GAGs, in addition to HP. It has a similar charge to HP and the other GAGs, but its structural background is built up of entirely different carbohydrates. The experiment was carried out in a 384-well plate format with vectors encoding firefly luciferase [20].

**Fig 2:**
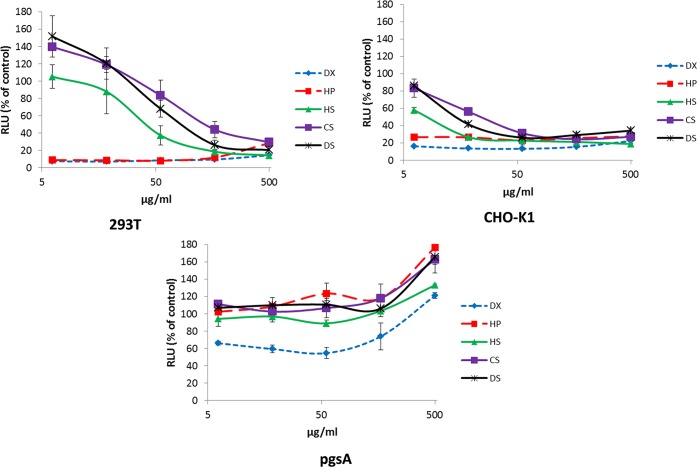
Transduction of cells with CHIKV Env pseudotyped vectors in the presence of soluble GAGs. 293T (left), CHO-K1 (right), and pgsA-745 (bottom) cells were seeded in 384-well plates and transduced with CHIKV Env-pseudotyped vectors transferring a *luciferase* gene. Before addition to the cells, the vector particles were incubated with DX or one of the indicated GAGs for 30 minutes at 4°C (DX and GAGs in five 3-fold dilutions, ranging from 500.0 to 6.2 μg/μl). One day after transduction, the luciferase expression of the cells was detected by a luminometer [20]. The results are given as percentages of the untreated control values. The experiment was carried out twice in triplicate, and one representative triplicate result is shown.

Transducing 293T and CHO-K1 cells with CHIKV Env-pseudotyped vectors transferring a luciferase gene in the presence of GAGs generally resulted in dose-dependently reduced transduction efficiencies compared to the control without GAGs (Fig 2, top). DX and HP were the most potent inhibitors on both cell lines. On 293T cells, the cell entry could be inhibited to about 10–20% of the untreated control at a concentration of 500 μg/μl GAGs. On CHO-K1 cells, transduction was reduced to about 20–30% of the GAG-free control maximally (Fig 2, top). Cell entry of CHIKV Env-pseudotyped vectors into pgsA-745 cells was, on the contrary, dose-dependently enhanced by the addition of rising GAG concentrations (Fig 2, bottom). The highest values reached, at 500 μg/μl, were between 121 (DX) and 177% (HP) of the untreated controls. Substantial inhibition of transduction with values lower than 100% was only observed for DX at concentrations lower than 500 μg/μl. In conclusion, the data indicate that cell entry of CHIKV Env-pseudotyped vectors is enhanced by GAGs, since low level transduction is still possible in pgsA-745 cells and in the presence of soluble GAGs. In addition, the entry into GAG-deficient pgsA-745 cells could be enhanced with increasing amounts of GAGs, indicating an activation of CHIKV Env-pseudotyped vectors.

**Fig 3:**
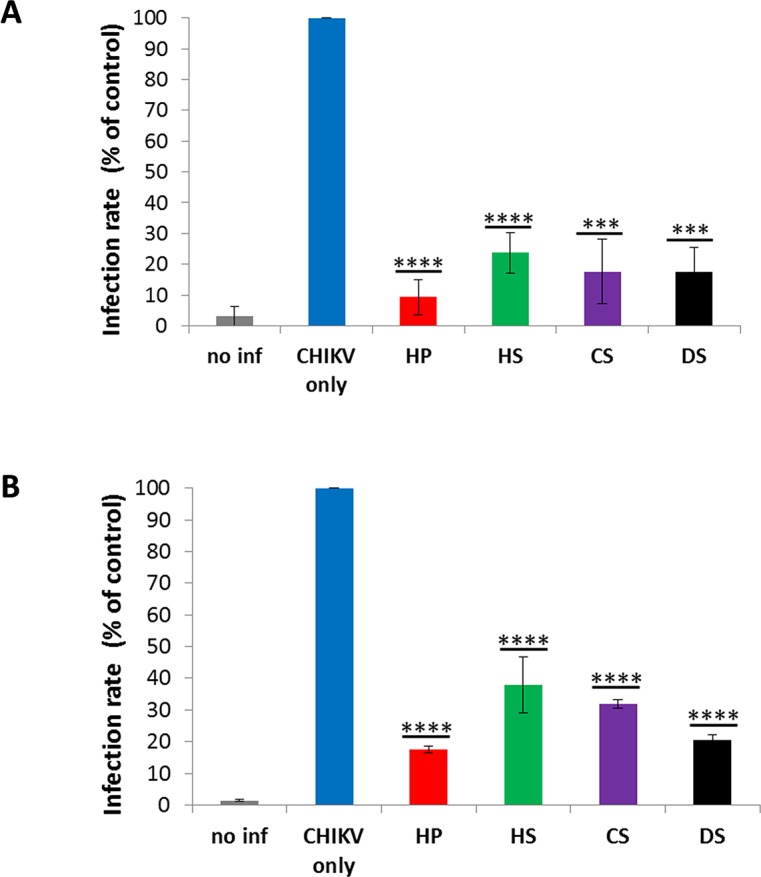
Infection and cell entry by CHIKV-mCherry-490 in the presence of soluble GAGs. A: 293T cells were seeded in 24-well plates and 500 μg/ml of the indicated GAG was added. Afterwards, cells were infected with CHIKV-mCherry-490 using an MOI of 1. The viral replication was determined 6 hrs post-infection by flow cytometry detecting mCherry. B: 500 μg/ml of the indicated GAG and CHIKV-mCherry-490 (MOI 1) were incubated together at 4°C for 30 minutes. After addition to 293T cells, another incubation of 30 minutes at 4°C followed. Then, the unbound virus together with the respective GAG were washed away and fresh medium was added to the cells. The viral replication was determined 6 hrs post-infection by flow cytometry detecting mCherry. Data represent the average of three independent experiments. *** and **** indicate significant differences in infection rates to untreated control cells. ** (P ≤ 0.01) and *** (P ≤ 0.001).

As shown above, CHIKV replication in GAG-deficient cells was diminished. Accordingly, this raised the question of whether infection of cells is inhibited in the presence of soluble GAGs. 293T cells were infected with CHIKV-mCherry (MOI 1) in the presence of 500 μg/ml of the respective soluble GAGs (but 500 U/ml HP). Six hours later, cells were analyzed by flow cytometry. Fig 3A shows that all GAGs reduced viral replication significantly by at least 76.3% (HS). HP was most effective, reducing replication by 90.7% compared to the GAG-free control. To determine whether the inhibition of CHIKV replication by GAGs occurs at the attachment/entry step of the viral life cycle, CHIKV and the different GAGs were incubated with target cells at 4°C to allow viral attachment to the cells but to avoid the subsequent endocytosis step. After 30 minutes, unbound virus and the GAGs were washed away and the cells were incubated for 6 hrs at 37°C in fresh medium. Infection rates were measured via flow cytometry. Fig 3B shows, similar to the previous experiment, a significant reduction of cell attachment/entry by 62.1 (HS) to 82.4% (HP).

In conclusion, replication of recombinant CHIKV-mCherry-490 in 293T cells is significantly inhibited by the addition of soluble GAGs. Attachment and/or endocytosis are the critical steps in the viral life cycle where this inhibition occurs.

### Cell binding properties of recombinant proteins expressed from *E*.*coli* consisting of E2 domain A, B, C, and the extracellular part of E2

**Fig 4:**
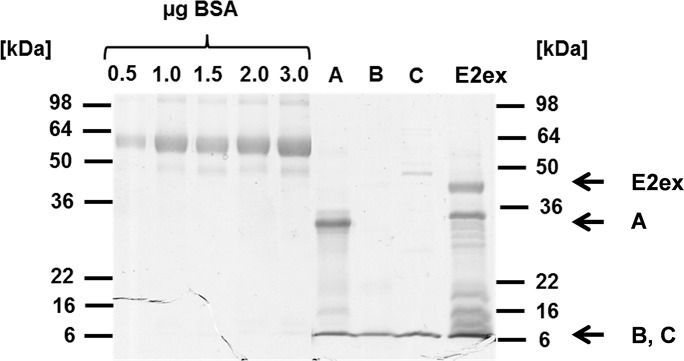
Purification of recombinant CHIKV E2 domains A, B, and C, and E2ex. The CHIKV E2 domains A, B, and C, and E2ex were cloned into the pET-15b vector, and the proteins were expressed in *E*. *coli* and purified under native (B, C) or denaturing conditions (A, E2) by HPLC (Ni-NTA beads). For A and E2, ion-exchange chromatography was additionally performed after the HPLC. Subsequently, they were dialyzed against PBS and concentrated. Shown is an SDS-PAGE with subsequent Coomassie staining of the purified proteins. BSA dilutions were used to estimate the protein concentration. Domain A has a molecular mass of 26.5 kDa, B of 8.5 kDa, C of 10.7 kDa, and E2ex of 40.4 kDa.

The E2 protein is the cell-binding moiety of CHIKV and has been frequently described to contain determinants recognized by neutralizing antibodies [7], [8], [25]. Therefore, one could speculate that parts of E2 are components that bind the unidentified cellular receptor of CHIKV. To analyze which domains of the E2 protein are involved in cell binding, the sequences encoding domain A (including the β-ribbon connector, aa 1–171 and 231–270), domain B (aa 172–231) and domain C (aa 271–341) were cloned into the bacterial expression vector pET-15b. The same was done for the entire extracellular part of the E2 protein (E2ex), which served as a positive control. The constructs also contained an N-terminal poly-histidine-tag for purification. The proteins were expressed in *E*. *coli*, and partially purified by Ni^2+^ affinity chromatography under native (E2 domains B and C) and denaturing (E2 domain A and E2ex) conditions. For domain A and E2ex additional ion-exchange chromatography was performed to remove bacterial protein contaminants. Fig 4 shows a Coomassie-stained SDS-PAGE separation of the purified proteins. Domain A has a molecular mass of 26.5 kDa, B of 8.5 kDa, C of 10.7 kDa, and E2ex of 40.4 kDa. The purified proteins migrated at the expected size.

**Fig 5:**
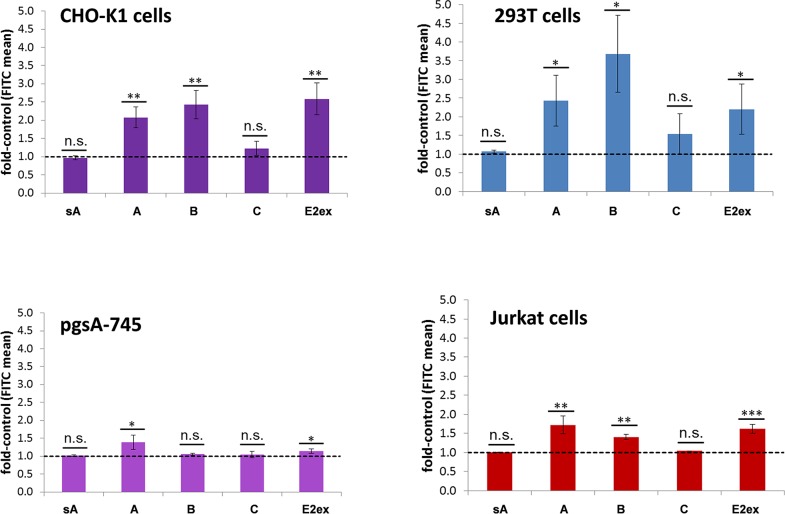
**Binding of recombinant CHIKV E2 domains A, B, and C, and E2ex to CHO-K1 and pgsA-745 cells.** CHO-K1 (top) and pgsA-745 (bottom) cells were incubated with 10 μg of the indicated recombinant proteins. Binding was measured by flow cytometry using an anti-His-tag and an anti-mouse FITC conjugated antibody. The results are shown relative to the control (anti-His-tag and anti-mouse FITC antibodies alone). A value of one represents the mean FITC value of the control and is labeled by the dashed line. Values above this line indicate binding. Data represent the average of three independent experiments. * and ** indicate significant differences to the recombinant protein-free controls. n.s. means not significant. * (P ≤ 0.05) and ** (P ≤ 0.01). Protein sA served as a negative control [23].

Their cell binding was analyzed with CHO-K1, the GAG deficient pgsA-745, 293T and Jurkat cells.293T cells show good transduction efficiencies with CHIKV Env-pseudotyped vectors. In contrast, Jurkat cells have revealed very low transduction efficiencies [20]. The recombinant proteins were incubated with cells at 4°C. Bound protein was detected by flow cytometry using an anti-His-tag antibody and an anti-mouse IgG-FITC antibody. Data are presented as fold increase in the mean FITC values of the sample in comparison to those of the control (cells only treated with staining antibodies). In addition, the protein sA, which contains only surface-exposed domains of A and did not induce neutralizing anti-E2 antibodies upon vaccination [23] and was used as a negative control for the binding studies. Fig 5 reveals that domains A and B, and protein E2ex showed a significant difference in the mean FITC signal compared to the control sample, indicating binding of these proteins to 293T and CHO-K1 cells. In contrast, there was no significant cell binding detectable for the negative control, sA, and domain C. An identical pattern was obtained for Jurkat cells, although at a generally lower binding level. Conducting the experiment with pgsA-745 cells resulted in weak, yet still significant, binding of domain A and E2ex, and no binding of domain B or C (Fig 5). Accordingly, these data indicate that binding of domain B to cells is enabled by cell-surface GAGs, while that of domain A is only partly dependent on these molecules.

**Fig 6:**
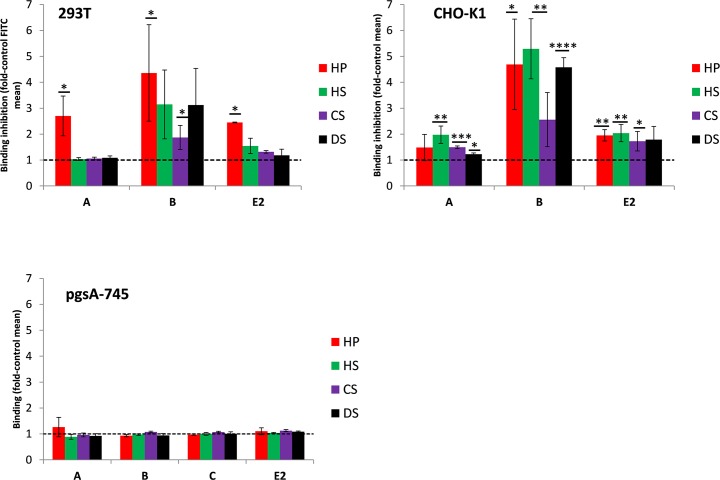
**Binding of recombinant CHIKV E2 protein domains A, B, and C, and E2ex to 293T, CHO-K1, and pgsA-745 cells in the presence of soluble glycosaminoglycans (GAGs).** 10 μg of the indicated recombinant proteins were incubated with the indicated soluble GAGs (500 μg/ml) for 30 minutes at 4°C. 293T (top), CHO-K1 (middle), and pgsA-745 (bottom) cells were then incubated with this mixture. Binding was measured by flow cytometry using an anti-His-tag and an anti-mouse FITC conjugated antibody. The results are shown as relative values to the control (incubation of cells with the respective recombinant protein alone). A value of one represents the mean FITC value of the control and is labeled by the dashed line. Values above this line indicate inhibition of binding. Data represent the average of three independent experiments. * and ** indicate significant differences to the GAG-free controls. n.s. means not significant. * (P ≤ 0.05), ** (P ≤ 0.01), *** (P ≤ 0.001) and **** (P ≤ 0.0001).

To further prove that cell binding of the E2 protein domains is GAG dependent, inhibition of their cell binding by addition of soluble GAGs was analyzed. The recombinant E2 proteins and different cell lines were again incubated at 4°C for binding; however, this time in the presence of 500 μg/ml soluble GAGs. Heparin (HP) was used as a control to ascertain the role of charge in the cell binding. HP is structurally derived from heparan sulfate (HS), but is more heavily sulfated and thus has a higher negative charge density. Fig 6 shows the results of soluble GAG-induced protein-binding inhibition and indicates that binding of domain A to 293T cells was not reduced in the presence of any of the soluble GAGs, except HP (about 2.5-fold). In contrast, the presence of all GAGs reduced the cell binding of domain B up to 3.0-fold. Again, HP showed the most efficient inhibition (4.5-fold). Inhibition of protein E2ex binding was in between that of domains A and B. A similar picture was observed for CHO-K1 cells (Fig 6, middle). Here, in contrast to 293T cells, HP did not strongly inhibit domain A binding. Minor inhibition of domain A binding was detectable after addition of HS and chondroitin sulfate (CS). The same experiment was performed with pgsA-745 cells (Fig 6, bottom). Here, domain C was used as an additional control. However, the binding of the recombinant proteins to pgsA-745 cells was not inhibited or enhanced in the presence of soluble GAGs. The viability of 293T cells incubated with 500 μg/ml of the different GAGs (the maximum concentration used in all experiments) was tested using MTT assays. None of the GAGs were significantly cytotoxic at this concentration (data not shown).

In summary, binding of E2 domain A to 293T and CHO-K1 cells was not or weakly inhibited by the addition of soluble GAGs. Only the strong negatively charged HP inhibited the interaction of domain A with 293T cells. In contrast, binding of domain B to both cell lines was massively decreased in the presence of HP, HS, CS, and dermatan sulfate (DS). The addition of GAGs to domains A, B, or C had no influence on the binding properties of these proteins towards GAG-deficient pgsA-745 cells.

### Expression of E2 domains as immunoglobulin Fc-fusion proteins and analysis of their cell binding activities

**Fig 7:**
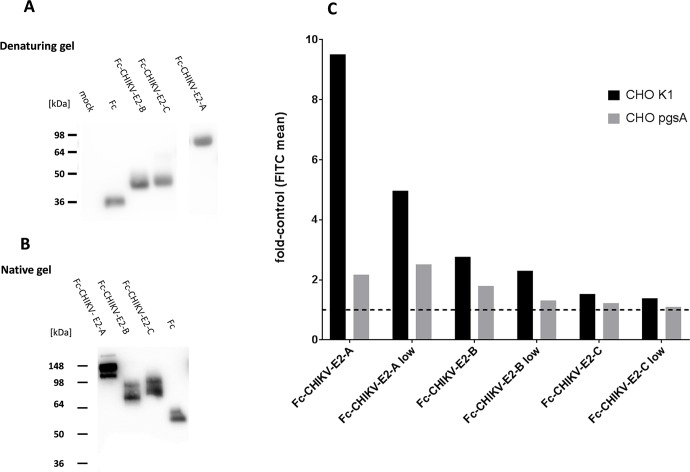
**Binding of recombinant Fc-fusion proteins containing CHIKV E2 domains A, B, and C to CHO-K1 and pgsA-745 cells.** A: Fc-E2-fusion proteins and Fc protein were expressed from HEK293T cells, affinity purified by protein-A chromatography and separated by SDS-PAGE under reducing conditions. The Western blot was detected with an HRP-labeled anti-human IgG antibody. The calculated molecular weights are: Fc 29.2 kDa; Fc-CHIKV-E2-B 35,0 kDa; Fc-CHIKV-E2-C 37.1 kDa and Fc-CHIKV-E2-A 53 kDa. B: Separation of the Fc-fusion proteins under native conditions. The Western blot was detected with an HRP-labeled anti-human IgG antibody. C: CHO-K1 (black) and pgsA-745 (grey) cells were incubated with the indicated recombinant Fc-fusion proteins as single measurements. Binding was measured by flow cytometry using an anti-human IgG FITC conjugated antibody. The results are shown as fold induction compared to Fc binding. Two volumes were use either 400 μl or 100 μl (low). A value of one represents the mean FITC value of the control and is labeled by the dashed line. Values above this line indicate binding. Data represent an average experiment of three independent experiments performed.

There was a serious concern that the E2 domains expressed in *E*. *coli* might not be correctly folded or have incorrect disulfide bond patterns. Therefore the E2 domains were expressed as Fc-fusion proteins in a soluble form in eukaryotic cells by transient transfections of 293T cells. Supernatants containing these proteins were partially purified by protein-A Sepharose chromatography and used for binding assays (Fig 7A). Western blot analysis of the proteins under native conditions confirmed that the proteins were Fc-dimers, as expected for antibodies (Fig 7B). This implies that disulfide bonds were formed and might be present not only in the Fc part, but also in the E2 fragments.

First the three subdomains A, B and C were tested for their cell binding ability as Fc-fusion proteins towards CHO-K1 and pgsA-745 cells. The Fc protein was used as negative control. Two concentrations were tested and “low” represents one sixth of the original sample. Again, domain A and B bound to CHO-K1 cells (Fig 7C). Domain C bound only marginally to CHO-K1 cells. This distribution is similar to the one determined with proteins expressed by *E*.*coli* (Fig 5), although domain A expressed from eukaryotic cells had a higher binding affinity compared to domain B, than domain A derived from *E*.*coli*. Analysis of cell binding to pgsA-745 cells revealed that domain A shows residual cell binding in the absence of GAGs. Domain C did not bind to pgsA-745 cells.

**Fig 8:**
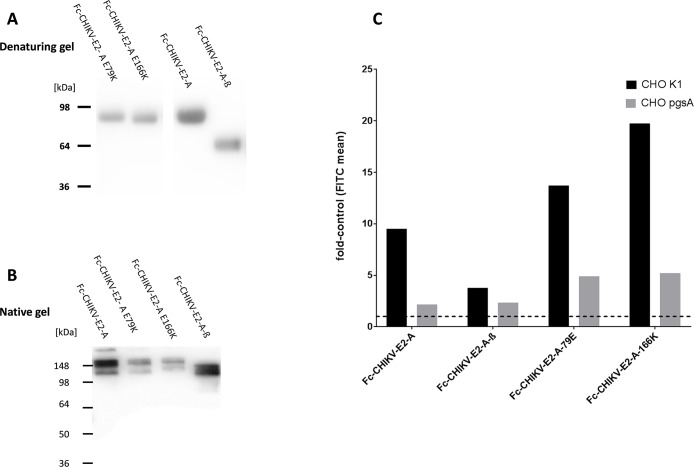
Binding of Fc-fusion proteins containing variants of the CHIKV E2 domain A to CHO-K1 and pgsA-745 cells. A: Fc-E2 domain A-fusion proteins and Fc protein were expressed from HEK293T cells, affinity purified by protein-A chromatography and separated by SDS-PAGE. The Western blot was detected with an HRP-labeled anti-human IgG antibody. The calculated molecular weights are: Fc-CHIKV-E2-A, E79K and E166K 53 kDa; Fc-CHIKV-E2-A-ß 43,4 kDa. B: Separation of the Fc-fusion proteins under native conditions. The Western blot was detected with an HRP-labeled anti-human IgG antibody. C: CHO-K1 (black) and pgsA-745 (grey) cells were incubated with the indicated recombinant Fc-fusion proteins. Binding was measured by flow cytometry using an anti-human IgG FITC conjugated antibody. The results are shown as fold induction compared to Fc binding. A value of one represents the mean FITC value of the control and is labeled by the dashed line. Values above this line indicate binding. Data represent an average experiment of three independent experiments performed.

Point mutations in domain A have been described before to increase CHIKV binding to GAGs [15], [16], [18], therefore we generated two mutants of domain A and expressed them as Fc-fusion proteins, domain A with a mutation at position 79 (Fc-CHIKV-E2-A-E79K) and position 166 (Fc-CHIKV-E2-A-166K). In addition, we generated a construct containing domain A without the ß-ribbon connector (A-ß) (Fig 8A). Again, the proteins run in SDS-PAGE under native conditions as dimers (Fig 8B). The two point mutants in domain A showed a higher binding affinity to CHO-K1 cells compared to domain A (Fig 8C) and their binding to pgsA-745 cells was only weakly increased. This indicates that introducing the point mutations in domain A increased its binding affinity to GAGs. Deletion of the ß-ribbon connector decreased domain A binding to CHO-K1 cells, however binding to pgsA-745 cells remained unaltered compared to domain A containing the ß-ribbon connector. This indicates that the ß-ribbon connector partially contributes to GAG binding and that the GAG independent binding site is located in domain A.

## Discussion

The alphavirus CHIKV enters cells by receptor-mediated endocytosis and a subsequent pH-dependent fusion step [26]. Host factors that are required for CHIKV entry are still only poorly understood. First hints have emerged from genome-wide RNAi screens, where downregulation of archain1, fuzzy homologue or TSPAN9 inhibited CHIKV infection but also that of alphaviruses in general [27]. On the viral side, the CHIKV E2 glycoprotein mediates cell attachment; however, the detailed mechanism has not been studied well so far [5], [6].

The structures of the CHIKV envelope proteins have been solved by X-ray crystallography [7], [8]. The extracellular part of E2 has three immunoglobulin (Ig)-like extracellular domains called A, B, and C [7]. Here, we expressed the extracellular part of E2 (E2ex) and its three domains A, B and C in *E*. *coli*. Cell-binding experiments revealed a significant binding of domains A and B, and E2ex, but not domain C, to all cells tested. Domain C is found close to the viral membrane and is followed by the stem region, which is a linker to the transmembrane region composed of hydrophobic amino acids [7]. Since domain C is not surface accessible and does not contain epitopes for neutralizing antibodies [7], it was not surprising that this protein lacked cell-binding activity. Domain B is located at the tip of the protein protruding from the viral surface and is linked to domain A via a long β-ribbon connector (containing an acid-sensitive region [ASR]). Domains A and B are prominently exposed on the viral membrane and it has previously been speculated that the cellular receptor mainly interacts with domain A [7,8,28]. The recombinant proteins containing domains A and B bound to cells independently of the cells’ ability to allow cell entry of CHIKV [20]. Binding to non-permissive Jurkat cells was reduced in comparison to binding to highly permissive 293T cells, which indicates that the binding moiety might be present on all cells but at different densities.

Further analysis of the possible cellular attachment factor was performed with the help of CHO-K1 and pgsA-745 cells. The pgsA-745 cells are derived from CHO-K1 cells and lack glycosaminoglycans (GAGs) on the cell surface. Binding assays revealed that domains A and B bound to CHO-K1, but only domain A bound to pgsA-745 cells, however markedly reduced but still significant. Furthermore binding of domain B to GAG-expressing cells in the presence of soluble GAGs was highly reduced. This suggests that domain B binds GAGs and thereby facilitates CHIKV cell binding. In contrast, domain A binding to GAG-deficient pgsA-745 cells and competition with soluble GAGs only marginally decreased domain A cell binding, indicating GAG-independence.

These data were confirmed with Fc-fusion proteins containing the E2 domains A, B and C, suggesting that the *E*.*coli* derived proteins have a native structure. In addition, point mutations in domain A showed the previously observed increase in GAG binding. Deletion of the ß-ribbon connector decreased the GAG binding of domain A, indicating that the ß-ribbon connector is partially responsible for GAG binding. A GAG binding consensus sequence is located in the ß-ribbon connector (-DRKGK- amino acid 251–255) [29]. GAG independent binding was not affected and might be mediated by domain A.

These data were substantiated by transduction of pgsA-745 cells with CHIKV Env-pseudotyped vector particles, which was significantly reduced by over 50% in comparison to transduction of the parental CHO-K1 cells, and soluble GAGs inhibited transduction of GAG-expressing cells. Remarkably, the transduction rates could not be reduced to less than 10% of the soluble GAG-free control, indicating that at least one GAG-independent entry pathway must exist. The presence of soluble GAGs did not decrease, but rather enhance transduction of pgsA-745 cells with CHIKV Env-pseudotyped vectors. This might be simply due to a local acidic environment generated by soluble GAGs, which may induce a fusion competent conformation of the glycoprotein. Alternatively, a pre-activation of the CHIKV envelope proteins through binding to the soluble GAGs might occur. Soluble GAGs did not enhance the binding of domains A or B to pgsA-745 cells; therefore, it is tempting to speculate that GAGs might induce conformational changes within the envelope proteins that allow them to bind more effectively to structures on the cell surface, which then might promote viral uptake. Such an activation of the virus has been described for the human papillomavirus type 16 (HPV-16) in the presence of HP, which allowed HPV-16 infection in the absence of cell-surface GAGs [30]. Cell-surface HS is an important attachment factor for HPV-16 [31]. For AAV-2 particles, slight structural rearrangements on the viral surface have been described upon HP binding [32]. Furthermore, it has been proposed that initial structural rearrangements on the alphavirus surface occur directly after binding to the cell surface [9], based on the observation that transitional epitopes of the Sindbis virus (SINV) became accessible to antibodies upon cell binding [33,34, 35].

Replication of CHIKV was also significantly reduced in pgsA-745 cells in comparison to CHO-K1 cells at 6 hrs post infection. However, this effect was less pronounced 24 hrs post infection, although still significant. CHIKV replication in 293T cells was significantly reduced in the presence of soluble GAGs. The lack of GAG-dependency at a later time point during infection might be explained by potential direct cell-to-cell transmission, as it has been observed for CHIKV in cell culture [36], or could just be due to the saturation of infection. For example, the majority of cells could already be infected 18 hrs post-infection and the viral titer might already be at a plateau, regardless of whether the cell entry is less efficient or not. However, an enhancement of infection by soluble GAGs was not observed. This might indicate that higher concentrations of GAGs are needed, which are possibly within their toxic range.

So far, the role of GAGs in CHIKV replication has mainly been studied with regard to viral attenuation. Point mutations within the E2 domain A (e.g., E79K, G82R or E166K) have been found in attenuated vaccine strains that were cell-culture adapted and showed enhanced GAG-dependency but reduced *in vivo* replication [15], [16], [18]. These mutations increase the positive charge in domain A [16], and as shown here for the first time, directly affect its binding affinity. In addition, recently it has been shown that CHIKV binding to GAG receptors on mammalian cells enhances replication in those cells and cell binding was influenced by the N-glycosylation pattern of the viral envelope proteins [37]. There are two glycosylation sites in E2, one in the ß-ribbon connector (N-263) and another one in the stem region, outside the region that was analyzed here. Deletion of the ß-ribbon connector and expression of domain A in E. coli decreased domain A’s cell binding, indicating that glycosylation of N-263 may partially contribute to GAG binding. In contrast to domain A, domain B has mostly been described to be associated with covering the E1 protein and thus antibodies binding to domain B mainly inhibit the movement of domain B during fusion, but not cell attachment [25]. Our findings reveal a unique role for cell-surface GAGs during CHIKV infection, in which they are not absolutely necessary for CHIKV replication, but undoubtedly promote viral entry and replication. There is at least one GAG-independent entry pathway, as CHIKV entry into GAG-deficient cells is still possible, and soluble GAGs cannot fully block CHIKV cell entry. This additional pathway(s) consequently include different cell surface receptor(s). The proposed entry pathways are most likely mediated by different binding sites on the E2 protein. The GAG-dependent pathway would be characterized by binding of the prominently exposed domain B most likely in combination with domain A to cell-surface GAGs, inducing a conformational change of the CHIKV Env molecules which might allosterically spread to the other CHIKV Env molecules on the particle surface [9]. This activation might enable the envelope molecules to bind to a second molecule on the cell surface, possibly via domain A. Following this binding, the virus is taken up by receptor-mediated endocytosis and the further pH-induced conformational changes occur. The proposed key role for domain B is supported by experiments with chimeric viruses which revealed that domain B is the critical target of neutralizing antibodies in humans and mice [38]. The second, GAG-independent pathway in this model possibly involves direct binding of domain A to another cell-surface molecule which might not play a role in the GAG-dependent pathway.

GAGs on the cell surface are thus not absolutely required for CHIKV cell entry, but they are part of a strategy CHIKV employs in order to enter cells. They might be comparable with T-cell immunoglobulin and mucin (TIM) membrane proteins that promote, although are not absolutely required for, the cell entry of a number of viruses, including CHIKV [39]. The fact that CHIKV is able to infect a wide range of, and evolutionary only distantly related, species, and to infect many different cell types and organs within one organism [6], makes the scenario plausible, in which the virus can utilize the ubiquitously expressed GAGs for entry [40], but additionally exploits other opportunities and receptors to get into the host cell. Finally, GAG mimetics might thus be promising antiviral candidates for the treatment of CHIKV infections however they will not inhibit the GAG-independent entry pathway [41,42]. Because the E2 domains B and A contain cell binding moieties, they might be promising targets for vaccine development [23].
